# Dynamical reorganization of the pluripotency transcription factors Oct4 and Sox2 during early differentiation of embryonic stem cells

**DOI:** 10.1038/s41598-020-62235-0

**Published:** 2020-03-23

**Authors:** Paula Verneri, Camila Vazquez Echegaray, Camila Oses, Martin Stortz, Alejandra Guberman, Valeria Levi

**Affiliations:** 1 CONICET - Universidad de Buenos Aires, Facultad de Ciencias Exactas y Naturales, Departamento de Química Biológica, Instituto de Química Biológica (IQUIBICEN), Buenos Aires, Argentina; 2 Universidad de Buenos Aires, Facultad de Ciencias Exactas y Naturales, Departamento de Fisiología y Biología Molecular y Celular, Buenos Aires, Argentina

**Keywords:** Pluripotent stem cells, Cellular imaging, Nuclear organization

## Abstract

Pluripotency maintenance requires transcription factors (TFs) that induce genes necessary to preserve the undifferentiated state and repress others involved in differentiation. Recent observations support that the heterogeneous distribution of TFs in the nucleus impacts on gene expression. Thus, it is essential to explore how TFs dynamically organize to fully understand their role in transcription regulation. Here, we examine the distribution of pluripotency TFs Oct4 and Sox2 in the nucleus of embryonic stem (ES) cells and inquire whether their organization changes during early differentiation stages preceding their downregulation. Using ES cells expressing Oct4-YPet or Sox2-YPet, we show that Oct4 and Sox2 partition between nucleoplasm and a few chromatin-dense *foci* which restructure after inducing differentiation by 2i/LIF withdrawal. Fluorescence correlation spectroscopy showed distinct changes in Oct4 and Sox2 dynamics after differentiation induction. Specifically, we detected an impairment of Oct4-chromatin interactions whereas Sox2 only showed slight variations in its short-lived, and probably more unspecific, interactions with chromatin. Our results reveal that differentiation cues trigger early changes of Oct4 and Sox2 nuclear distributions that also include modifications in TF-chromatin interactions. This dynamical reorganization precedes Oct4 and Sox2 downregulation and may contribute to modulate their function at early differentiation stages.

## Introduction

Pluripotent stem cells self-renew and differentiate to all cell types derived from the three germ layers in response to developmental cues. These cells are an excellent model to study embryo development and development-related disorders, could also be used for drug screening and constitute an important promise in regenerative medicine^1,2^.

The maintenance of pluripotency depends mostly on three transcription factors (TFs) namely Oct4 (also known as Pou5f1), Sox2 and Nanog which induce genes necessary to preserve the undifferentiated state and repress others involved in differentiation^3^. Knockout of any of these TFs leads to embryo lethality and loss of pluripotency^4–7^.

Although these TFs coordinately regulate a great number of genes, their expression profiles are different during differentiation. Particularly, Nanog concentration rapidly decreases during the transition from the ground state to primed pluripotency while Oct4 and Sox2 levels remain constant both in cultured embryonic stem (ES) cells and in the embryo^8^. These observations emphasize that the modulation of the activity of Oct4 and Sox2 during these early differentiation stages involves other mechanisms.

The expansion and optimization of methodologies aimed to visualize nuclear processes in living cells revealed that transcription is a complex process with multiple spatial and temporal layers of control^9^. Chromatin architecture per se is an important player on the modulation of gene expression. For example, ES cells differentiation and embryo development are associated to a reorganization of chromatin that includes homologous pairing of Oct4 alleles^10^.

The hierarchically folding of chromatin within the nuclear space also defines regions with different levels of compaction that modulate TFs diffusion and the target-searching process^11^. Additionally, epigenetic marks locally regulate the accessibility of TFs to their targets modifying the transcriptional outcome^12,13^.

TFs interact dynamically with a wide variety of chromatin targets; their distribution among all these sites also impacts on the transcriptional output^14^. In line with this statement, we have found variations on chromatin-Sox2 interactions among blastomeres of 4-cell mouse embryos that correlate with the cell fate of the progeny^15^.

Furthermore, many transcription-related biomolecules partition into compartments which are not enclosed by membranes^16^ and contribute to buffer the amount of molecules in the nucleoplasm^17,18^. Very recently, it has been proposed that a liquid-liquid phase separation process drives the formation of many nuclear compartments^19^ that may play important roles in the local remodeling of chromatin^20^ and genes activation^21,22^ finally impacting on the transcriptional output^23^. Notably, Oct4 is required for the formation of Mediator liquid condensates in ES cells^21^. Additionally, it was proposed that Mediator foci occur at super-enhancers in ES cells and it was suggested that the recruitment of transcription-related molecules at these condensates modulates transcription of genes key for cell identity^24^.

These previous works show that the regulation of transcription and thus cell decisions cannot be completely understood by just analyzing the nuclear concentration of transcription-related molecules. Here, we use a combination of advanced fluorescence microscopy techniques in live cells to explore how Oct4 and Sox2 distribute in the nucleus of ES cells and inquire whether this organization changes during early differentiation. Our experiments showed that Oct4 and Sox2 redistribute in the nuclear space as early as 12–24 h after inducing differentiation by 2i/LIF withdrawal. We also detected that chromatin-TFs interactions respond to early differentiation cues. These results evidence a dynamical reorganization of these TFs that may impact on their functions at early differentiation stages preceding their downregulation. These observations provide valuable clues to understand the molecular mechanisms involved in cell fate decisions.

## Results

### Generation of mouse embryonic stem cell lines with doxycycline-inducible expression of YPet-tagged Oct4 or Sox2

We first generated ES cell lines that express Oct4 or Sox2 fused to the fluorescent protein YPet in an inducible manner. This system allows controlling the expression levels of the fluorescent TFs and minimizes the large cell-to-cell variability observed in transient transfections^25^. Additionally, the expression of the TFs-YPet can be turned on when desired, which is essential for many of the experiments described below. A previous work shows that the fluorescent TFs present similar genome-wide binding profiles to those obtained for the untagged TFs and that the expression of the fluorescently-tagged TFs rescues pluripotency of Oct4 or Sox2 knockout ES cells^26^.

**Figure 1:**
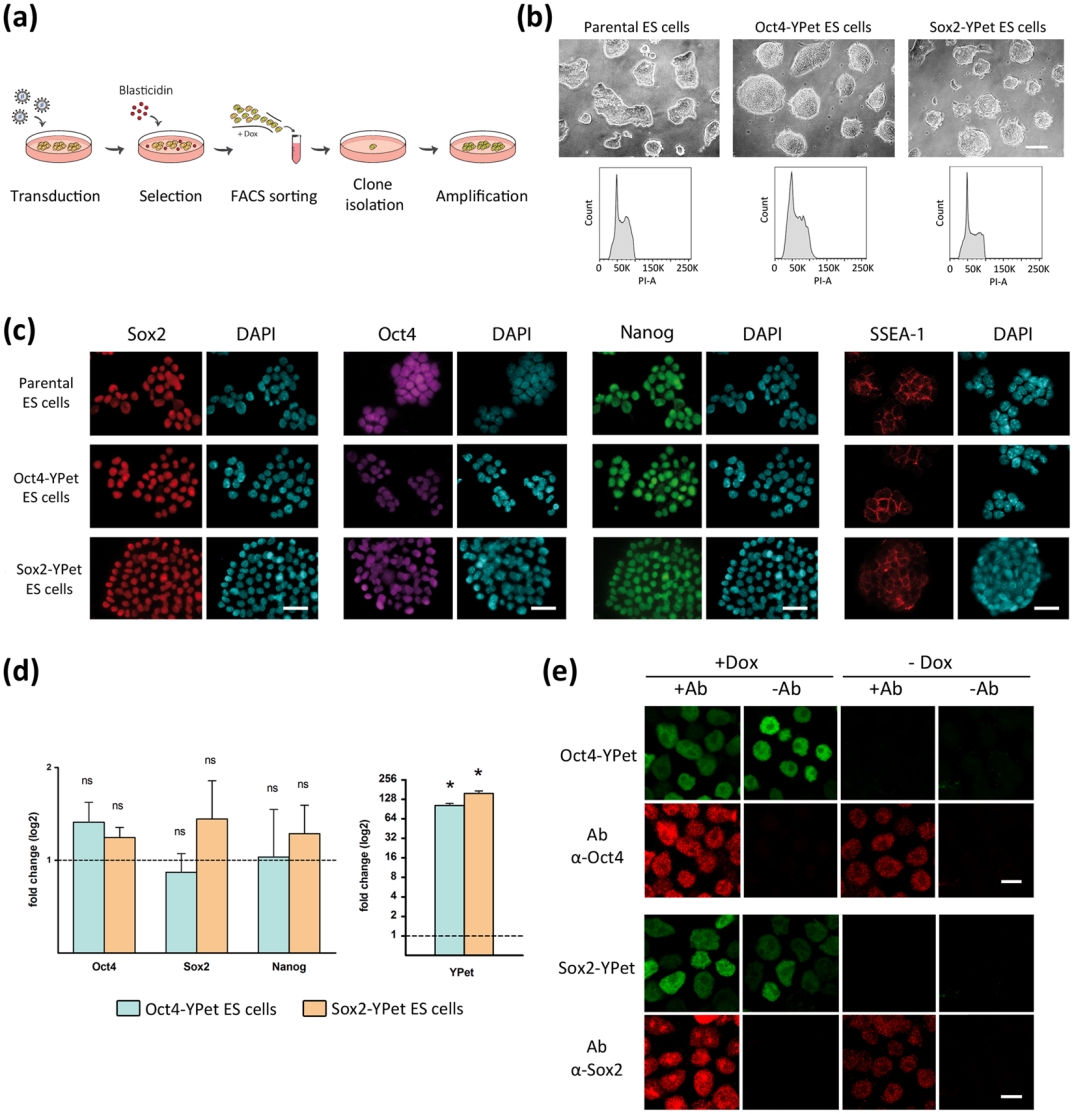
Generation and characterization of inducible ES cell lines. **(a)** Diagram of the experimental protocol used for the establishment of the stable ES cell lines Oct4-YPet and Sox2-YPet. **(b)** Representative phase contrast images showing the characteristic morphology of ES cell colonies and the cell cycle distribution of the parental, Oct4-YPet and Sox2-YPet ES cell lines after propidium iodide staining and flow cytometry analysis. Scale bar, 120 μm. **(c)** Representative immunostainings of the pluripotency markers Sox2, Oct4, Nanog and SSEA-1 in the parental cells, compared to Oct4-YPet and Sox2-YPet ES cells cultured in propagation medium in basal conditions (without Dox). Nuclei were counterstained with DAPI. Sox2 and Nanog proteins were co-immunostained. Scale bar, 30 μm. Intensity quantification is shown in Supplementary Fig. S1. **(d)** RT-qPCR analysis of the indicated genes in Oct4-YPet and Sox2-YPet ES cells cultured in propagation medium in the presence of Dox. Results are presented as means ± SEM (n = 3) and plotted in log2 scale. Data were relativized to the basal condition (without Dox, dotted line). No significant differences were detected in the expression of the analyzed genes comparing induced versus non-induced conditions, except for the TF-YPet fusion transgene in both cell lines (p = 0.0004). **(e)** Representative confocal images showing the distribution of Oct4-YPet and Oct4 proteins in Oct4-YPet ES cells (upper panel), or Sox2-YPet and Sox2 proteins in Sox2-YPet ES cells (lower panel), cultured in propagation medium. Immunostainings were performed in the presence (+Ab) or absence (−Ab) of the primary antibody, and cells were cultured for 48 h with (+Dox) or without (−Dox) Dox. Scale bar, 10 μm.

Briefly, we transduced W4 ES cells following the lentiviral-based strategy described in Materials and Methods and schematized in Fig. 1a. After 15 days of blasticidin selection, YPet positive cells were selected by fluorescence-activated cell sorting (FACS) from the doxycycline (Dox) induced cell population. Isolated single cells were amplified and the selected clones expressing fusion proteins were analyzed in detail for their validation.

Figure 1b shows brightfield images of the selected single clones (Oct4-YPet and Sox2-YPet) that were YPet positive in Dox-induced conditions (see below) and presented the expected colony morphology and normal cell cycle. Additionally, these clones expressed similar amounts of the pluripotency markers Sox2, Oct4, Nanog and SSEA-1 in comparison to the parental cell line as assessed by immunofluorescence (Fig. 1c and Supplementary Fig. S1).

To set the optimal conditions for Dox-induction, we analyzed widefield fluorescence images of the generated clones as a function of Dox concentration or time of induction (Supplementary Fig. S2). Based on these results, we decided to induce the expression of these fusion proteins by incubating the cells with 5 μg/ml Dox for 48 h. This condition guaranteed adequate fluorescence intensity levels for the experiments described in the following sections.

After Dox induction, the generated clones presented mRNA levels of Oct4 or Sox2 slightly higher than those of the parental ES cell line (Fig. 1d). Western blot analyses showed that the intensity of the Oct4-YPet band was ∼10% of that corresponding to the endogenous Oct4 (Supplementary Fig. S1b) suggesting that the total levels of this TF did not greatly increase after Dox induction. Unfortunately, the Sox2 antibody did not recognize Sox2-YPet (Supplementary Fig. S1b); therefore, we used fluorescence correlation spectroscopy (FCS) to determine the concentration of this fusion protein^27^. Using the procedure detailed in Methods, we estimated that Sox2-YPet concentration in the nucleoplasm of undifferentiated Sox2-YPet ES cells was 0.63 ± 0.03 µM (N_cells_ = 110) which is lower than the concentration of the endogenous protein^28^. Moreover, Dox treatment did not modify Nanog mRNA levels. Since this TF is a well-known gene target of both Oct4 and Sox2, this result suggests that the low Dox-induced expression of Oct4 and Sox2 do not considerably modify ES cells gene expression (Fig. 1d). Additionally, the subcellular distributions of these fluorescent fusion proteins and the corresponding endogenous TFs were the same (Fig. 1e) in line with a previous report showing that YPet fusion does not affect TFs localization^29^.

Altogether, these results support that the expression of Oct4-YPet and Sox2-YPet did not alter substantially the profile of pluripotency markers.

### Nuclear reorganization of Oct4 and Sox2 at the onset of differentiation

**Figure 2:**
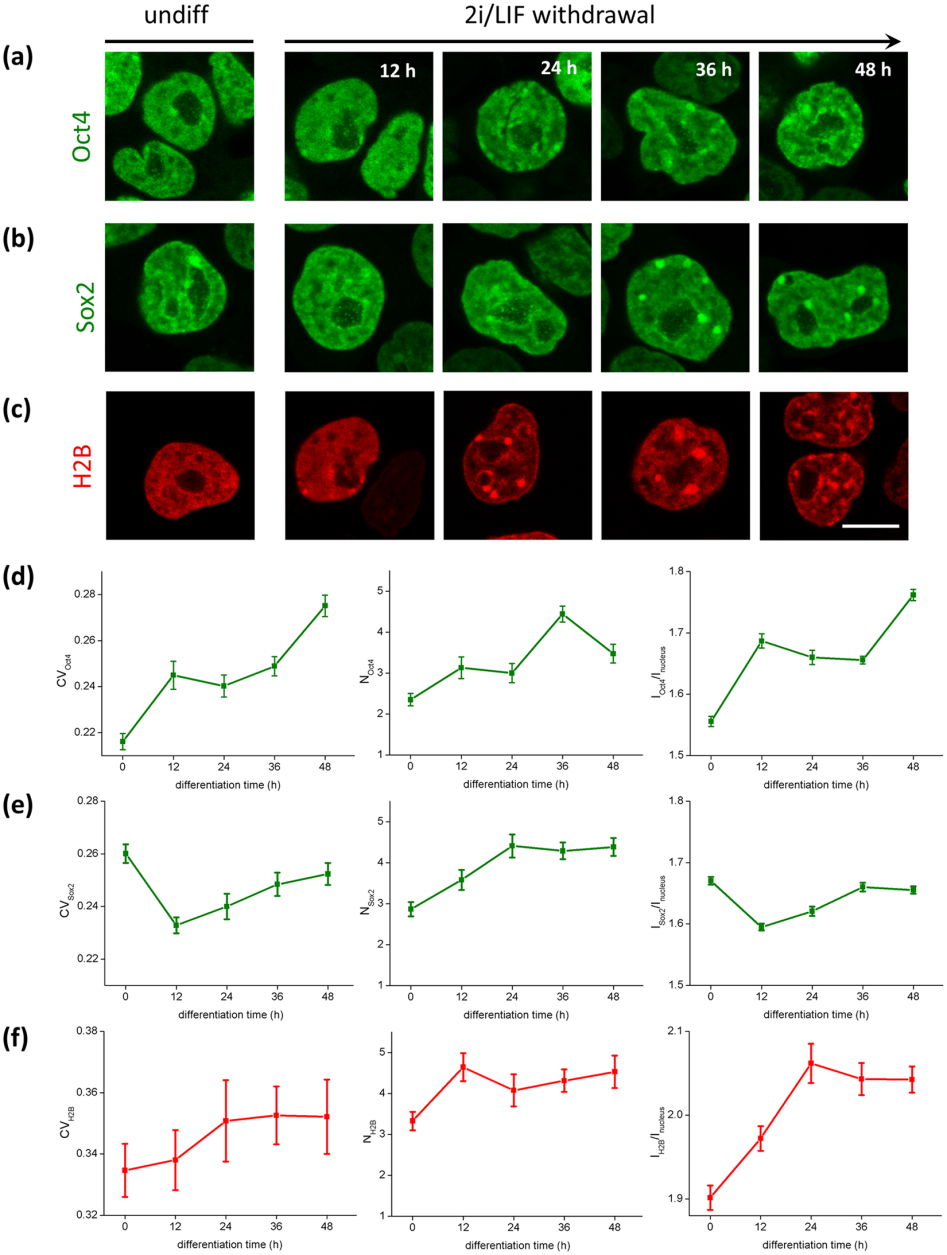
Reorganization of chromatin and Oct4 and Sox2 during early differentiation. Representative confocal images of Oct4-YPet ES cells expressing Oct4-YPet **(a)**, Sox2-YPet **(b)** or H2B-mCherry **(c)** as a function of time after 2i/LIF withdrawal. Scale bar, 10 μm. The nuclear distribution of these proteins was quantitatively analyzed as described in the text to obtain the coefficient of variation (CV), the number of dense domains (N) and their intensity relative to the mean nuclear intensity (I/I_nucleus_) for **(d)** Oct4-YPet, **(e)** Sox2-YPet and **(f)** H2B-mCherry. Results are presented as means ± SEM. N_cells_ were 106, 62, 67, 138 and 99 (Oct4-YPet); 140, 79, 73, 104 and 141 (Sox2-YPet); 58, 59, 40, 48 and 49 (H2B-mCherry) for 0, 12, 24, 36 and 48 h, respectively.

Figure 2a shows that Oct4-YPet partitions between the nucleoplasm and a few brighter domains or *foci* in the nucleus of ES cells cultured in the presence of LIF + 2i (“undifferentiated cells”). These observations agree with previous results suggesting that this TF is included in liquid condensates in ES cells^21^. Interestingly, Sox2-YPet also forms *foci* similar to those observed for Oct4-YPet (Fig. 2b).

In order to analyze if there is a correlation between *foci* formation and the local compaction of chromatin, we also examined the distribution of the histone H2B fused to mCherry in undifferentiated cells. Figure 2c shows that H2B-mCherry displayed a few concentrated domains as reported previously^30–32^; these domains colocalized with regions enriched in HP1α−EGFP (Supplementary Fig. S3), a protein commonly associated with silenced heterochromatin regions^33^.

Supplementary Fig. S4 shows that Sox2 and Oct4 *foci* colocalized with regions of condensed chromatin. The relative intensities of TF-YPet and H2B-mCherry in single cells varied among *foci* suggesting that the local compaction of chromatin is not the only factor determining the recruitment of TFs at these domains.

To explore if the spatial organization of Oct4 and Sox2 changes during the initial stages of differentiation, we induced differentiation of Oct4-YPet and Sox2-YPet ES cells by 2i/LIF withdrawal and registered confocal images during the initial 48 h of the differentiation process. Supplementary Fig. S5 shows that the endogenous levels of Oct4 and Sox2 in the parental cell line remain constant during these early stages of differentiation whereas Nanog expression significantly decreases in this time window, in line with previous observations^8^. The evident changes observed in cell morphology together with the downregulation of the naïve pluripotency markers Nanog, Klf4 and Esrrb and the increase of the mRNA levels of primed state-associated markers Oct6, Dnmt3a and Otx2 (Supplementary Fig. S6) evidenced that ES cells leave behind the ground state of pluripotency.

We quantified the intensity distribution of the TFs-YPet at early stages of the differentiation process determining the coefficient of variation (CV_TF_), which has been previously used to measure the overall distribution of nuclear proteins^34,35^, the mean number of bright *foci* per nucleus (N_TF_) and their intensity relative to that of the nucleus (I_TF_/I_nucleus_). Figure 2d shows that CV_Oct4,_ N_Oct4_ and I_Oct4_ increased as early as 12–24 h after 2i/LIF withdrawal reflecting the formation of *foci* enriched in Oct4. The redistribution of Sox2 at the onset of differentiation was different to that observed for Oct4. Particularly, Fig. 2e shows an initial reduction in CV_Sox2_ and I_Sox2_ concomitant to an increase of N_sox2_ within the first 12 h of differentiation suggesting that this TF redistributes from a few and bright *foci* to a higher number of dimmer *foci* in this early stage. After this period, the values of the parameters increased reflecting a further reorganization of Sox2 toward the formation of many *foci*. As a control, we repeated these analyses in independently generated clones of Oct4-YPet and Sox2-YPet and found that the studied parameters followed similar behaviors to those described above (Supplementary Fig. S7). We should mention that we detected some variations in the parameter values between clones that could be due to slightly different measurements conditions. Nevertheless, these small differences do not affect the main conclusions.

We also characterized the changes occurring in the large-scale organization of chromatin within this time window of the differentiation protocol. Figure 2c shows that the overall organization of chromatin changed towards a more heterogeneous and speckled distribution. The number of bright domains and their relative intensity increased while CV_H2B_ exhibited a tendency to increase within the first 24 h of differentiation (Fig. 2f), revealing an extensive chromatin remodeling in this time window. After this period, the parameters did not present major changes suggesting the absence of large-scale chromatin reorganization sensed through H2B-mCherry fluorescence.

The comparison of Fig. 2d–f shows that the distribution of TFs changed even after those parameters characterizing H2B-mCherry distribution remained constant. This observation suggests that TFs redistribution during early differentiation involves other mechanisms besides the chromatin reorganization previously discussed.

Taken together, these results suggest that Oct4 and Sox2 reorganize within the nuclear space at the onset of differentiation despite their concentrations do not change significantly during these early stages.

### Dynamical distribution of TFs in the nucleus at the onset of differentiation

We previously mentioned that the dynamical distribution of TFs among different compartments within the nuclear space might impact on transcription. Therefore, we used single-point fluorescence correlation spectroscopy to analyze the dynamics of Oct4 and Sox2 in the nucleus of undifferentiated ES cells and in cells cultured during 48 h in the absence of 2i/LIF (“early-differentiated cells”).

**Figure 3:**
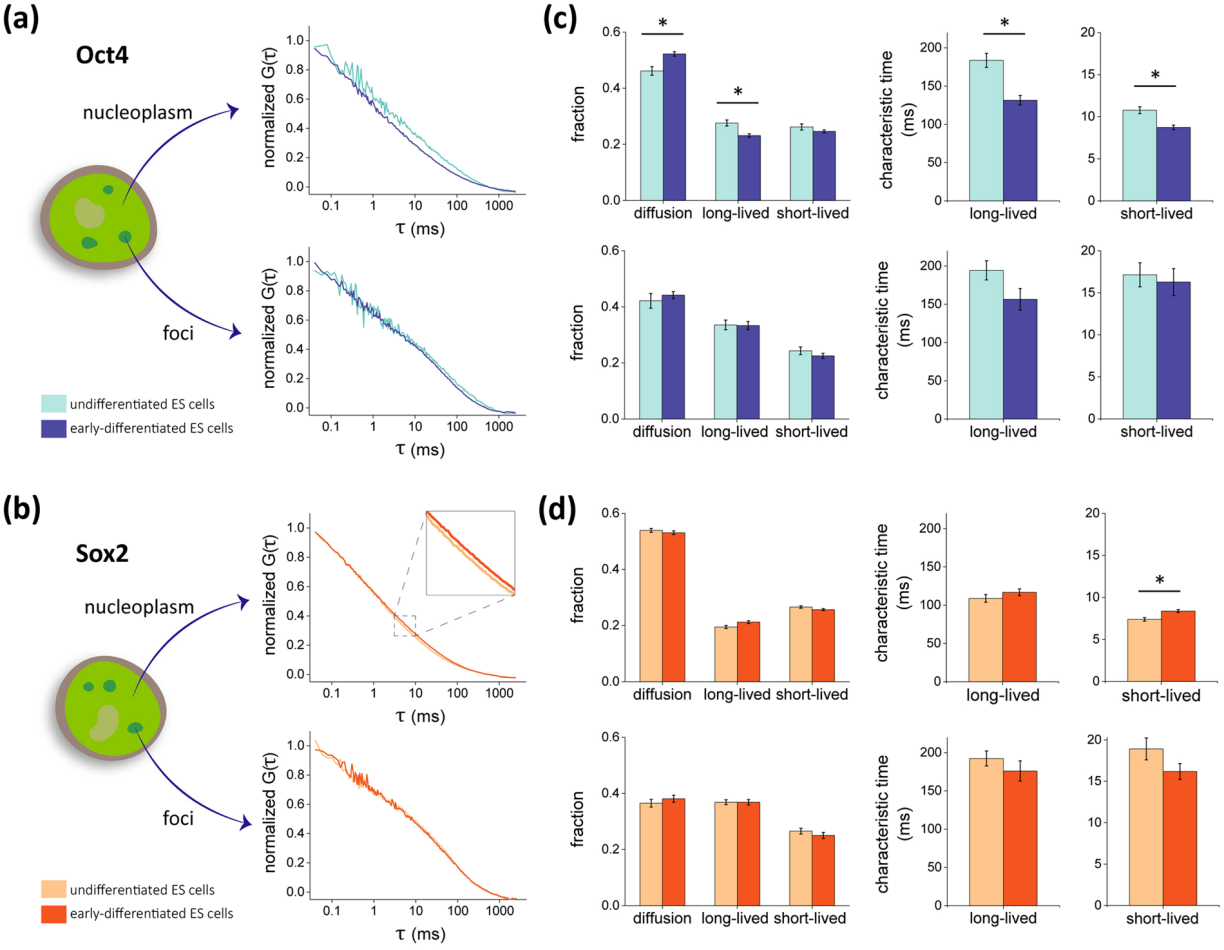
Fluorescence correlation spectroscopy reveals changes on Oct4 and Sox2 interactions with chromatin at the onset of differentiation. Single-point FCS measurements were run in undifferentiated Oct4-YPet and Sox2-YPet ES cells (light colors) or 48 h after LIF/2i withdrawal (early-differentiated, dark colors). **(a,b)** Mean, normalized ACF obtained at the nucleoplasm (top panel) and *foci* (bottom panel). **(c,d)** The ACF data were fitted with Eq. 1 to obtain the fractions of free, long-lived bound and short-lived bound TFs and the characteristic times of long-lived and short-lived interactions of the TFs with chromatin. Experimental results were expressed as mean ± SEM (FCS experiments in the nucleoplasm: n_data Oct4_ = 54 and 73 and n_data Sox2_ = 110 and 146 for the undifferentiated and early-differentiated conditions, respectively; FCS experiments at *foci*: n_data Oct4_ = 15 and 19 and n_data Sox2_ = 20 and 14 for the undifferentiated and early-differentiated conditions, respectively). We only run a single experiment in each cell to minimize its photodamage. Asterisks (*) indicate significant differences (p < 0.01) between undifferentiated and early-differentiated ES cells.

Figure 3a,b show the mean, normalized autocorrelation functions (ACF) obtained for Oct4-YPet and Sox2-YPet in undifferentiated and early-differentiated ES cells. The ACF curves could be interpreted with a model that includes the diffusion of the proteins and their interactions with chromatin targets in two distinct temporal windows^15,18,36^. The fitting of the model to the experimental data provides relevant information on how TFs distribute within the nuclear space. The data shows that TFs molecules are engaged in long- and short-lived interactions with characteristic times of ∼150 and ∼10 ms, respectively (Fig. 3c,d).

FCS analyses also revealed differences in the dynamics of the TFs in the nucleoplasm of undifferentiated or early-differentiated cells. Figure 3c,d show that Oct4 detached from long-lived chromatin targets after 48 h of LIF/2i withdrawal. Additionally, the lifetime of short- and long-lived interactions with chromatin decreased also indicating impaired interactions in early-differentiated cells. On the other hand, Sox2 presented subtler changes in its dynamics. Particularly, the relative amounts of this TF engaged in short-lived and long-lived interactions did not change significantly, neither the lifetime of long-lived interactions. However, we did detect a slight increase in the lifetime of short-lived Sox2-chromatin interactions that probably includes transient, unspecific interactions involved in the target-searching process as discussed below. FCS experiments performed in independently generated clones revealed similar behaviors of the TFs-YPet in response to differentiation cues (Supplementary Fig. S8) supporting that the observations described above are inherent to the TFs and not related to specific properties of the clones.

In the previous section, we showed that Oct4 and Sox2 presented distinct patterns of nuclear reorganization within the first 12–24 h of differentiation (Fig. 2). Therefore, we quantified the dynamics of these TFs in the nucleoplasm at relevant time points included in this window. Supplementary Fig. S9a,c show that the dynamics of Oct4 after 24 h of differentiation was similar to that observed for undifferentiated cells suggesting that the changes in Oct4-chromatin interactions described above occur after longer differentiation times. Since we showed before that the CV of Sox2 presented a minimum value after 12 h from 2i/LIF withdrawal (Fig. 2c), we performed the FCS experiments at this particular time point. Supplementary Fig. S9b,d show that the dynamics of Sox2 after 12 h of differentiation is similar to that observed for early-differentiated cells. Specifically, the only change revealed by the FCS analyses was the slight increase in the lifetime of short-lived Sox2-chromatin interactions.

We also measured through FCS the dynamics of Oct4-YPet and Sox2-YPet at the high-intensity *foci* reported in the previous section. Figure 3 and Supplementary Fig. S10 show that Oct4 and Sox2 are engaged in longer interactions in these domains in comparison to those detected in the nucleoplasm. We should mention that the correlation curves did not relax in ∼40% (Oct4-YPet) and ∼20% (Sox2-YPet) of the acquired data suggesting that TFs at *foci* are probably involved in slower interactions beyond the time window assayed in the FCS experiments.

The FCS analyses revealed that the dynamics of Oct4 and Sox2 within *foci* did not present major changes after 48 h of 2i/LIF withdrawal suggesting that differentiation cues did not modify the interactions of TFs at these nuclear domains. These results indicate that differentiation induces the formation of a higher number of high-intensity TFs-enriched *foci* (Fig. 2) but does not modify the interaction landscape of the TFs in these domains.

## Discussion

The core pluripotency transcription factors play a fundamental role in the maintenance of stem cell pluripotency and in the repression of their differentiation.

Early works linked cell fate with changes in the levels of transcription factors measured either in bulk assays or in fixed cells or specimens. However, the development of new methods, some of them designed to observe transcription-related molecules in living systems at a single-cell level, revealed new aspects of transcription regulation and improved our understanding of this important process. For example, we now know that even small endogenous fluctuations in the levels of Oct4 and Sox2 influence cell fate commitment^26^.

The fascinating structure of the nucleus, comprising a variety of membrane-less open compartments with specific biochemical and physical properties^37^, determines a heterogeneous distribution of transcription-related proteins such as TFs^38^ and their tortuous diffusion within the nuclear space^11^. These properties ultimately define the probability of TFs encounters with their chromatin targets and therefore, may impact on transcription^11^.

In this context, we investigated the nuclear distribution and dynamics of the key pluripotency TFs Oct4 and Sox2 at early stages of differentiation in which their levels remain constant.

We generated stable ES cell lines that express either Oct4 or Sox2 fused to the fluorescent protein YPet in an inducible manner. The morphology of the colonies and the profile of pluripotency markers of these cell lines were similar to that of the parental ES cell line.

We found that Oct4 and Sox2 in undifferentiated cells distribute between the nucleoplasm and a few concentrated domains. These *foci* could act as reservoirs buffering the concentration of TFs in the nucleoplasm and therefore dampening fluctuations that may affect pluripotency maintenance. Remarkably, ES cells must maintain fine-tuned levels of Oct4 and Sox2 within narrow limits to preserve pluripotency^39^. In this direction, it was reported that Oct4 is included in liquid condensates containing the coactivator Mediator^21^, a complex that is located at super-enhancers in ES cells^24^. Moreover, Oct4 residues required for phase separation with Mediator are also needed for gene induction^21^, suggesting a link between these structures and TF function.

Very recently, it has been proposed that unspecific interactions with chromatin may also contribute to the formation of biomolecular condensates in the nucleus^40^. In line with this statement, we observed a colocalization of TFs *foci* with dense chromatin regions and longer lifetimes of short-lived TF-chromatin interactions at *foci* compared to the nucleoplasm. These observations suggest that unspecific TF-chromatin interactions may be involved in *foci* formation.

We also showed that, during the initial 12–24 h of differentiation induced by 2i/LIF withdrawal, chromatin reorganizes forming heterochromatinic, H2B-concentrated domains as observed in previous reports at longer differentiation periods^30–32^. This reorganization could also be related to the epigenetic reprogramming that occurs at the exit of the naïve state (reviewed in^41^) and probably influences the transcriptional output in line with evidence showing that the genome of ES cells is hyperactive and differentiation involves a large-scale silencing^42^.

Additionally, we found that Oct4 and Sox2 also reorganize in the nuclear space during early-differentiation. In the case of Oct4, TF molecules concentrate into a larger number of brighter *foci*. On the other hand, Sox2 forms dimmer *foci* within the first 12 h of differentiation and redistributes into a higher number of *foci* afterwards. Notably, the nuclear concentration of both Oct4 and Sox2 do not change in the assayed temporal window, i.e. 48 h, indicating that differentiation cues induce a different, fast reorganization of Oct4 and Sox2 in the nuclear space before their downregulation.

We also characterized how Oct4 and Sox2 organization changes at the onset of differentiation by analyzing their dynamics through FCS. This exquisite technique measures the dynamics of molecules with minimal photodamage and at the level of single cells. Additionally, the method has the advantage of requiring relatively low levels of fluorescent proteins minimally modifying the cell homeostasis. We have recently used this powerful tool to explore how certain epigenetic modifications in ES cells modulate Oct4 and Nanog interactions with chromatin^36^.

Here, we found that the dynamics of Oct4 and Sox2 followed a model including the diffusion of TFs and their engagement in fast (∼10 ms) and slow (∼150 ms) interactions with chromatin targets in agreement with our previous work^15^. These interactions probably involves unspecific binding, short-distance sliding on DNA, hopping^14,28,43–45^ and long-lived and more specific binding^46^. The faster interactions determine how the TFs move within the complex nuclear space and thus, how they find the specific targets^47,48^.

FCS detected variations in Oct4 and Sox2 interactions with chromatin targets during the initial stages of differentiation. While Oct4 interactions with chromatin weaken after 48 of 2i/LIF withdrawal, Sox2 only showed a slight increase in the lifetime of short-lived interactions after differentiation. FCS analyses also revealed that Oct4 and Sox2 interactions at *foci* did not change during early differentiation indicating that this process triggers the formation of a higher number of *foci* but with similar properties in terms of TF binding.

Relevantly, Oct4 and Sox2 dynamical distributions did not respond similarly to differentiation cues. Further evidences support differences in the molecular mechanisms involved in their function. For example, Hogan *et al*.^10^ observed that chromatin reorganization during early differentiation includes pairing of Oct4 but not Sox2 gene *loci*; this process is associated to an accumulation of the epigenetic mark H3K9me2 at Oct4 enhancer leading to the repression of this TF. Additionally, Strebinger *et al*.^26^ recently reported that the accessibility of enhancers associated to differentiation-promoting genes in ES cells increases in cells with high levels of Oct4 and do not correlate with Sox2 levels. Moreover, the transition between naïve and primed pluripotency involves a genome wide Oct4 relocalization triggered by the prime-state associated TF Otx2^49^.

We speculate that Oct4 and Sox2 dynamical changes found at early stages of differentiation are directly linked to modulation of the expression of specific genes. In this direction, we observed that some Oct4 and Sox2 targets associated to pluripotency maintenance such as Nanog, Klf4 and Esrrb^50^ are downregulated whereas differentiation–associated markers like Dnmt3a, Oct6 and Otx2 are upregulated within this time window. Transcription of this last TF is regulated by Oct4; once induced, Otx2 together with Oct4 regulate the expression of multiple genes leading to the initiation of differentiation^51^.

The exit from naïve pluripotency precedes the upregulation of lineage-associated genes and recapitulates early stages of embryo development^8^. Therefore, the dynamical changes of Oct4 and Sox2 observed in our work are probably related to the transition from the naïve to the formative pluripotency state. In this direction, we speculate that similar changes would also occur at early time windows of other differentiation protocols.

We should emphasize that the relation between TFs organization and gene regulation is very complex and far from understood. As mentioned above, a growing number of evidence supports that the nucleus behaves as a multi-phase compartment with a dynamic partition of nuclear biomolecules between distinct liquid phases^19^. As a consequence, nuclear compartmentalization may buffer the concentration of biomolecules in the nucleoplasm^52–54^, also increasing or reducing the concentrations of specific biomolecules in liquid domains, thus regulating the kinetics and specificity of the biochemical reactions occurring within these condensates^19,52,55^. These previous works show that changes in the distribution of a given TF probably affect a whole network of interactions involving TFs, chromatin and other transcription-related biomolecules. Therefore, further research in the field is required to understand how changes in the spatial distribution of TFs impact on gene expression.

**Figure 4:**
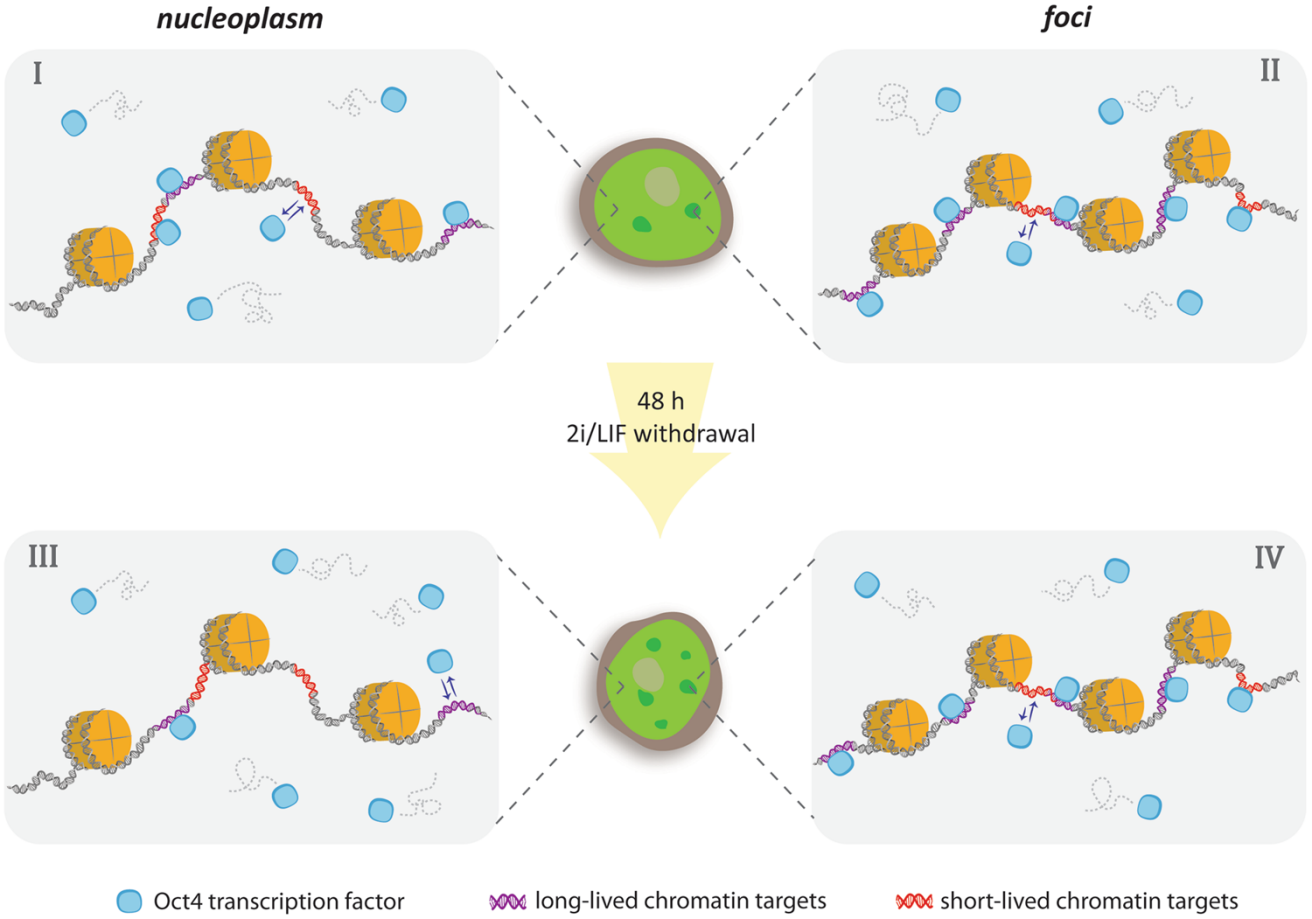
Cartoon of Oct4 dynamics in nuclei of undifferentiated or early-differentiated cells. To simplify the scheme, we represented a bead-on-string structure of chromatin and did not illustrate in the cartoon differences in Oct4-chromatin interactions lifetimes. Oct4 diffusing molecules engage in short-lived and long-lived interactions with chromatin in the nucleoplasm (I) or at chromatin-dense *foci* (II). Short-lived interactions last longer in these structures. 2i/LIF withdrawal triggers the formation of a higher number of *foci* (central panel). In the nucleoplasm of early-differentiated cells, the short- and long-lived lifetimes of Oct4-chromatin interactions decrease while the proportion of freely diffusing Oct4 molecules increases (III). In contrast, the dynamics of the TF does not change at *foci* (IV).

Figure 4 compiles some of the observations performed in this work; we only represent the dynamics of Oct4 to simplify the scheme.

In the undifferentiated state, Oct4 molecules diffuse within the nucleus and may interact with a variety of chromatin targets (I); additionally, TFs may cluster in *foci* (II) that colocalize with compacted chromatin; the interactions of TFs at *foci* last longer than those observed at the nucleoplasm.

During the initial stages of differentiation, Oct4 interact weaker with chromatin targets in the nucleoplasm (III) probably due to the modifications in the chromatin organization. Additionally, new Oct4 *foci* form during differentiation. Whereas the chromatin interaction landscape of Oct4 at each *focus* does not change (IV), the new *foci* sequester a higher amount of TF molecules. As a consequence, the number of Oct4 molecules available for interactions with specific chromatin targets diminishes probably modulating the transcriptional output.

Taken together, our results reveal that early differentiation cues trigger a dynamical reorganization of Oct4 and Sox2 within the nuclear space before their downregulation. Considering that the dynamical distribution of TFs between nuclear reservoirs define how these molecules find their specific targets on chromatin, their redistribution at early differentiation stages could impact on gene expression.

## Methods

### Cell culture and differentiation

The parental, mouse ES cell line (W4) was provided by the Rockefeller University Core Facility. ES cells were maintained on 0.1% gelatin-coated dishes, passaged every 3 days using trypsin (Gibco), and grown at 37 °C in a 5% CO_2_ (v/v) incubator. Cells were cultured in DMEM (Gibco) supplemented with 15% ES-cell qualified fetal bovine serum (Gibco), 100 mM MEM nonessential amino acids (Gibco), 2mM l-alanyl-L-glutamine (Gibco), 0.5 mM 2-mercaptoethanol, 100 U/mL penicillin (Gibco), 100 mg/mL streptomycin (Gibco), 1000 u/ml leukemia inhibitory factor (LIF), 1 μM PD0325901 (Tocris) and 3 μM CHIR99021 (Tocris), hereafter named propagation medium. HEK 293 T cells (ATCC) were cultured in DMEM supplemented with 10% fetal bovine serum (Internegocios S.A.), 100 U/mL penicillin (Gibco), 100 mg/mL streptomycin (Gibco). To induce non-directed differentiation, ES cells were cultured in the absence of LIF and 2i during the periods of time indicated in the text.

### Lentiviral transduction and generation of stable cell lines

Lentiviral vector production was carried out by PEI (Poliscience Inc.) transfection of HEK 293 T cells with the envelope plasmid psPAX2 (Addgene #12260) and packaging pMD2.G plasmid (Addgene #12259), together with the lentiviral vectors of interest (pLV-PGK-rtTA3G-IRES-bsd, pLVTRE3G-YPet-Sox2, pLVTRE3G-YPet-Oct4)^29^, kindly provided by Dr. David Suter (Institute of Bioengineering, Ecole Polytechnique Fédérale de Lausanne, Lausanne, Switzerland). Lentiviral particles-containing supernatants were collected 48 and 72 h after transfection, filtered through a 0.45 μm syringe filter and supplemented with 7 μg/ml polybrene (Sigma). Target parental ES cells were seeded at ∼100,000 cells per well of a 6-well plate and incubated for 24 h. Then, cells were transduced twice every 24 h with 1 ml of each fresh lentiviral supernatants collected as described above. After each round, cells were spinfected for 45 min at 750 xg. Finally, cells were incubated for 24 h and the medium was replaced with propagation medium. Selection of transduced cells with blasticidin antibiotic started 48 h after transduction at a concentration of 8 μg/ml and maintained for 15 days, with a medium change every other day. To enrich the population of cells with those containing the YPet Dox-inducible transgene, cells were sorted by flow cytometry in a FACS Aria II flow cytometer (BD Biosciences), collected, disaggregated to single cells and plated at a density low enough to grow as isolated colonies. Clone isolation was performed manually with a needle, picking the colonies and replating them separately in a 24-well plate. After amplification of each clone, validation was performed by cell cycle analysis, immunostaining and RT-qPCR. Finally, two clones were selected and gave rise to the corresponding ES cell lines denominated Oct4-YPet and Sox2-YPet. After performing the dose and time response curves described in the results section, doxycycline was used at a final concentration of 5 μg/ml for 48 h to perform all the experiments.

### Cell transfection

For transient expression of H2B-Cherry and HP1α-EGFP, cells were plated during 24 h onto coverslips coated with PDL and laminin as described above. Transfection was carried out using Lipofectamine 2000 (Thermo Fisher) and 1.6 µg of plasmid DNA in Opti-MEM medium (Thermo Fisher). The transfection medium was replaced after 24 h with fresh culture medium. Microscopy observations were performed 48 h after transfection.

### Cell cycle analysis

DNA content was analyzed as previously described^56^. Briefly, single cell suspensions were fixed in 70% ethanol, rehydrated in PBS and stained with 25 μg/ml Propidium Iodide (Sigma). After 30 min incubation, samples were analyzed in a FACS Aria II flow cytometer (BD Biosciences). Data was compiled using FlowJo software.

### Immunostaining

For immunofluorescence experiments, cells were grown on coverslips coated with Geltrex (Thermo Fisher) and cultured for at least 48 h. Cells were fixed with 4% paraformaldehyde, permeabilized with 0.1% Triton X-100 PBS-Tween 20 (PBST) and blocked with 10% donkey serum (Sigma) in PBST. Primary antibodies for Nanog (Peptrotech), Oct4 (Santa Cruz), Sox2 (Santa Cruz) and SSEA-1 (Santa Cruz) in block solution were added to the samples, incubated at 4 °C overnight and then washed 3 times with PBST for 10 min. Secondary antibodies (Invitrogen) and DAPI in block solution were added and incubated at room temperature for 1 h. Samples were washed as described before and mounted on slides with Mowiol. The antibodies used are listed in Supplementary Table S1.

### Quantitative Real-Time PCR (RT-qPCR)

RT-qPCR was performed and analyzed as previously described^56^, with minor modifications. Briefly, total RNA was extracted with QuickZol (Kalium Technologies) following manufacturer’s instructions and reverse transcribed using RevertAid Reverse Transcriptase (Thermo Fisher). Quantitative Real time PCR amplification of cDNA was carried out using FastStart SYBR Green Master (Roche) in a LineGene 9600 engine (BioER). Sequences for all primers used in qPCR analysis are listed in Supplementary Table S2. At least 2 biological replicates were performed in all the experiments, with 2 technical replicates for each condition. Gene expression was normalized to the geometrical mean of Gapdh and Pgk1 housekeeping values.

### Cells preparation for imaging experiments

For microscopy measurements, 18-mm round coverslips were placed into the wells of a 12-multiwell plate, incubated for 1 h with a 100 μg/ml Poly-D-Lysine (PDL) (Sigma) and 2 h with a solution 20 μg/ml laminin (Thermo Fisher) at 37 °C in a 5% CO_2_ incubator. Next, 70,000 cells were added in each well and incubated with culture medium. Doxycycline was added 48 h before observation.

### Fluorescence microscopy

Inmunofluorescence images were acquired in a widefield Olympus IX71 microscope (Olympus) equipped with an EXi Aqua Bio-Imaging Microscopy Camera (Qimaging).

Confocal experiments were run in a FV1000 Olympus microscope (Olympus). YPet and GFP fusion proteins were visualized using a multi-line Ar laser tuned at 488 nm as excitation source, whereas a 543 nm He-Ne laser was used for mCherry fusion proteins (average powers at the sample ∼1 µW). The laser light was reflected with dichroic mirrors 405/408 or DM405/488/543/635 for single-color and dual-color experiments, respectively and focused through an Olympus UPLSAPO 60X oil immersion objective (NA = 1.35) into the sample. Fluorescence was collected in the range of 500–600 nm (single-color experiments) or split into two channels set to collect between 500–530 nm and 600–700 nm (dual-color experiments).

### Fluorescence correlation spectroscopy

Single-point FCS measurements were performed in the FV1000 microscope described above, set in the pseudo photon-counting mode. The laser was focused at a position in a cell nucleus selected by the user and the intensity was collected at 50000 Hz during ∼3 min. We only run a single experiment in each cell to minimize its photodamage.

ACF data were calculated using SimFCS program (LFD, Irvine, CA, USA) and were fitted with Eq. (1) that considers the diffusion of the TFs and their binding to two populations of fixed sites^15^:

1 $${\rm{G}}({\rm{\tau }})=\frac{1}{{2}^{3/2}{\rm{N}}}\left[{{\rm{f}}}_{{\rm{D}}}{\left(1+\frac{{\rm{\tau }}}{{{\rm{\tau }}}_{{\rm{D}}}}\right)}^{-1}{\left(1+\frac{{\rm{\tau }}}{{{\rm{\omega }}}^{2}{{\rm{\tau }}}_{{\rm{D}}}}\right)}^{-1/2}+{{\rm{f}}}_{{\rm{short}}}{{\rm{e}}}^{-{\rm{\tau }}/{{\rm{\tau }}}_{{\rm{short}}}}+{{\rm{f}}}_{{\rm{long}}}{{\rm{e}}}^{-{\rm{\tau }}/{{\rm{\tau }}}_{{\rm{long}}}}\right]$$

where N is the mean number of fluorescent molecules in the confocal volume, τ_D_ is the characteristic diffusion time, ω is the ratio between axial and radial waists of the observation volume, and f_D_ is the freely diffusing population fraction. f_short_ and f_long_ are the population fractions bound to short-lived and long-lived targets, and τ_short_ and τ_long_ are their residence times, respectively. The reciprocal of the residence time corresponds to the dissociation constant k_off_.

### Image analyses

The coefficient of variation (CV) was calculated for each nucleus image as the ratio between the standard deviation (SD) of the nucleus intensity and the mean intensity of the nucleus. Nucleoli were not considered in these calculations since H2B-mCherry and TFs-YPet are excluded from these nuclear structures.

*Foci* were identified in images of nuclei binarized considering an intensity threshold equal to the mean intensity + 2.SD. The number, size and mean intensity of these foci were then calculated using the ImageJ plugin “Analyze Particles”. We only considered those structures with sizes ≥optical resolution.

### Estimation of concentration of YPet-TFs relative to the endogenous proteins

The proportion of Oct4-YPet relative to endogenous Oct4 was estimated quantifying the intensity of the corresponding bands in Western blot membranes using the ImageJ Gel Analyzer plugin. We should mention that this approach only provides an estimation of the relative protein levels since the affinity of the antibody for the fusion and endogenous proteins could be different.

The concentration of Sox2-YPet was estimated using point-FCS^27^ and compared to the endogenous levels reported in the literature. Briefly, the mean concentration of fluorescent molecules was calculated as γ/(G_o_V_conf_), where G_o_ is the value of G extrapolated to τ→0, V_conf_ = π ^3/2^ ω_x,y_^2^ ω_z_/√8 is the observation volume^57^ and γ is a geometric factor equal to 0.35 for a confocal setup.

### Statistical analysis

Experimental results were expressed as means + SEM of at least three biological replicates. Statistical significance between groups was analyzed using Lineal Mixed Models (LMM) followed by comparisons between means using the Tukey test, when required. Differences were regarded as significant at p ≤ 0.01. Residuals fitted normal distribution and homogeneity of variance. Otherwise, transformation of data was applied in some cases to meet both assumptions.

Comparison between the parameters presented in Supplementary Fig. S9 was performed using randomly-selected subsets of each parameter at the nucleoplasm to generate data sets with similar sizes to that collected at *foci*. Statistical tests showed identical results with all the subsets.

Statistical analysis was performed using either Infostat^58^ or the gls package of RStudio.

## Supplementary information

Supplementary Information.
