# Supplementary material for: Dynamical reorganization of the pluripotency transcription factors Oct4 and Sox2 during early differentiation of embryonic stem cells
Source: Sci Rep. 2020 Mar 23;10:5195. doi: 10.1038/s41598-020-62235-0 (PMC7089971; doi:10.1038/s41598-020-62235-0)
Supplement: Supplementary file 1 — Supplementary Information. [file 41598_2020_62235_MOESM1_ESM.pdf]

Dynamical reorganization of the pluripotency transcription factors Oct4 and Sox2 during early differentiation of embryonic stem cells

Paula Verneri, Camila Vazquez Echegaray, Camila Oses, Martin Stortz, Alejandra Guberman and Valeria Levi

1. Supplementary Figures

2. Supplementary Methods

3. Supplementary Tables

4. Supplementary References

Supplementary Figures

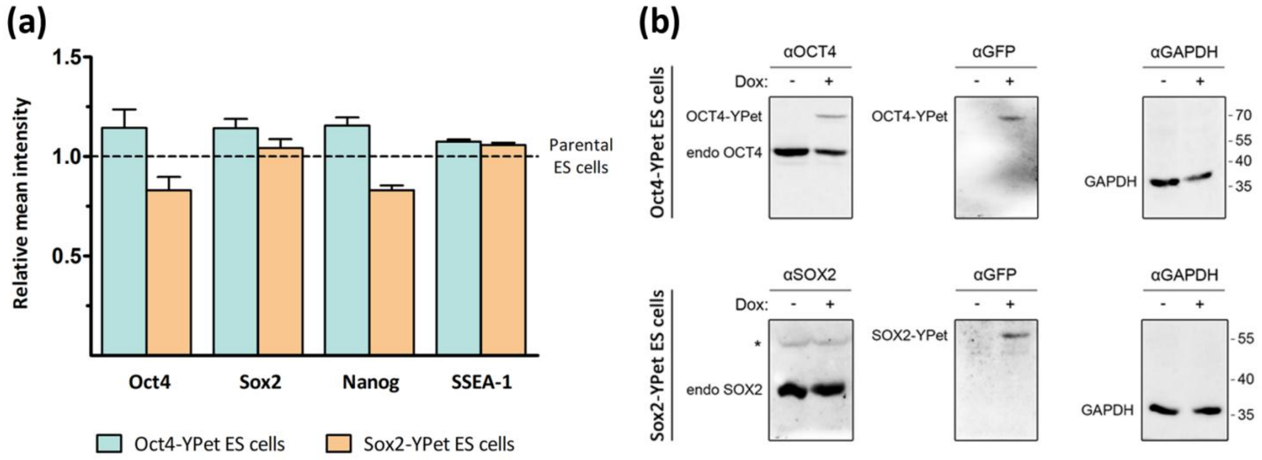

Figure S1. Characterization of Oct4-YPet and Sox2-YPet ES cell lines. (a) Oct4-YPet and Sox2-YPet ES cells present levels of the pluripotency markers similar than the parental ES cell line. Immunostaining of the pluripotency markers Sox2, Oct4, Nanog and SSEA-1 in the parental cells, compared to Oct4-YPet and Sox2-YPet ES cells, both cultured in propagation medium in basal conditions. Representative pictures are shown in Fig. 1c. The fluorescence intensity was quantified with ImageJ software. Bars represent the mean fluorescence intensity ± SEM relative to the corresponding mean intensity of the parental cells (N_{cells} ~150-200 cells from two independent experiments). No significant differences were found between Oct4-YPet or Sox-YPet ES cells and the parental cell line ($p<0.01$). **(b)** Immunoblots for whole cell lysates in the absence (-) or presence (+) of Dox, for Oct4-YPet ES cells (upper panels) and Sox2-YPet ES cells (lower panels). Primary antibodies used for each experiment are indicated above each panel (αOCT4, αSOX2, αGFP and αGAPDH). Identified proteins are listed to the left of each band, including the fusion proteins and the endogenous TFs (endo OCT4 and endo SOX2). GAPDH was included as a total mass control. The asterisk marks the position of a band corresponding to non-specific staining and/or Sox2 complexes. The positions and molecular weights of protein markers determined from the PageRuler protein Ladder (Thermo Fisher) are also stated in the right panels. For unknown reasons, the anti-Sox2 antibody did not interact with Sox2-YPet in the Western blot´s conditions and thus, we used an anti-GFP affinity-purified polyclonal antibody [1] for YPet protein recognition. Despite the antibody was raised against GFP, many anti-GFP antibodies also recognize YPet [2-4] since they have a high sequence homology [5,6]. In this direction, the top panel shows that this antibody also recognized Oct4-YPet.

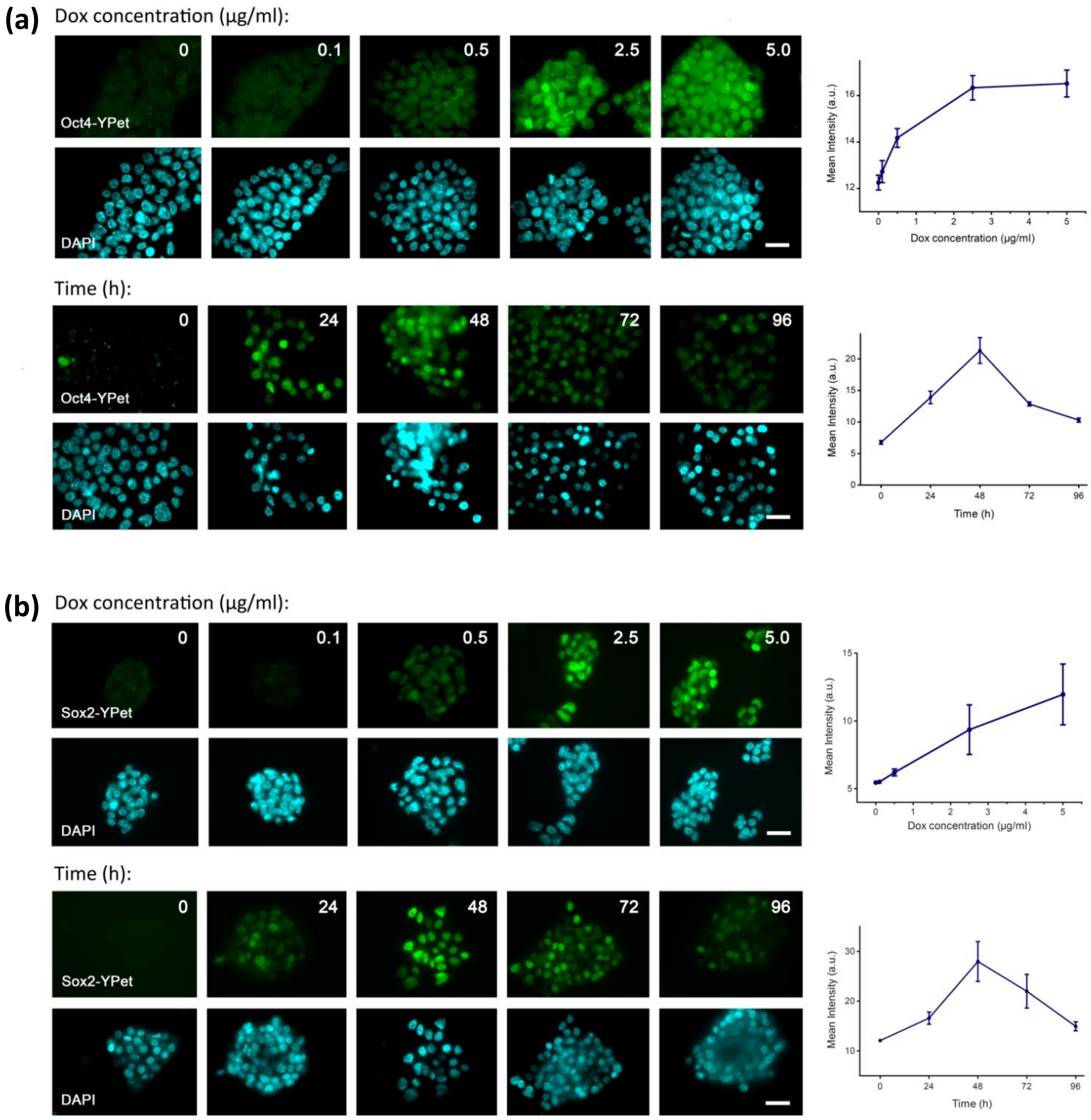

Figure S2. Optimization of Dox-induction protocol. Representative widefield fluorescence images of **(a)** Oct4-YPet and **(b)** Sox2-YPet ES cell colonies at different Dox concentrations or incubation times. For Dox concentration curves, the incubation time was 48 h whereas a Dox concentration of 5 μg/ml was used for the time-dependence experiments. Five different concentrations (0-5 μg/ml) and incubation times (0-96 h) were tested. Scale bar, 20 μm. A treatment of 5 μg/ml Dox for 48 h was selected for further experiments.

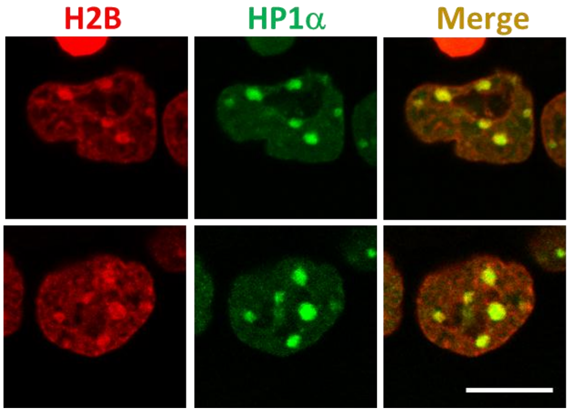

Figure S3. H2B-mCherry domains colocalize with regions enriched in HP1α-EGFP. Representative confocal images of parental ES cell line co-transfected with vectors encoding H2B-mCherry and HP1α-EGFP. Scale bar, 10 µm.

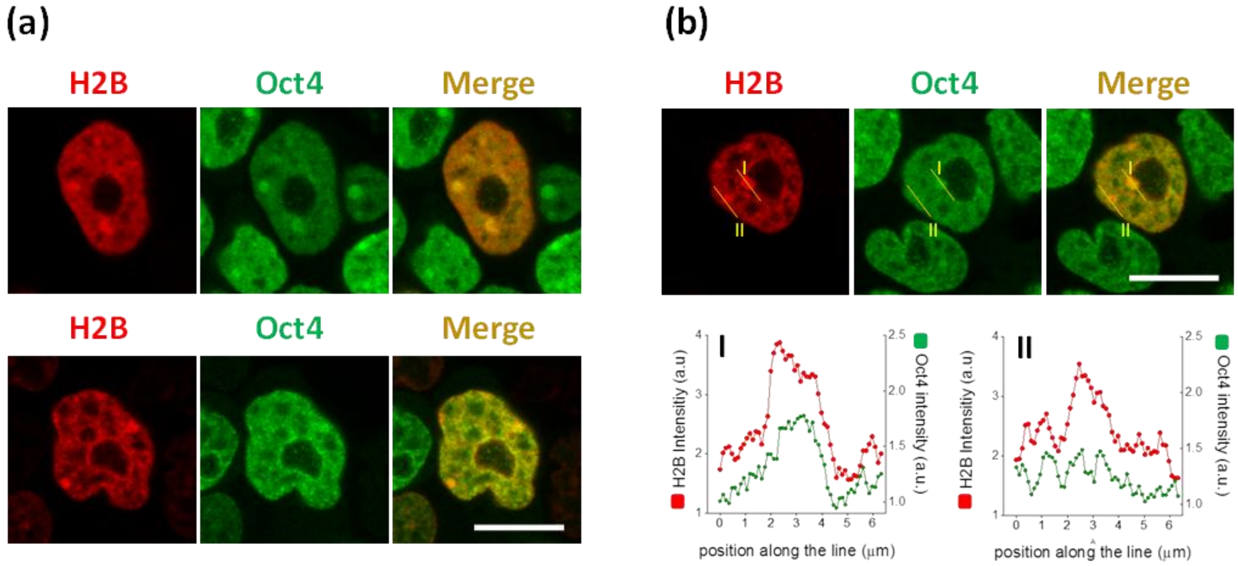

Figure S4. High-intensity TFs *foci* colocalize with mCherry-H2B domains. (a) Representative confocal images of Oct4-YPet (top) and Sox2-YPet (bottom) ES cells transiently transfected with H2B-mCherry. (b) The intensity profile along line I shows an H2B-mCherry enriched domain colocalizing with an Oct4-YPet high-intensity region whereas line II shows an H2B concentrated region that is not associated to a high Oct4-YPet intensity. While Oct4 concentrate in H2B-mCherry enriched regions, these regions do not always present high Oct4 intensity. Scale bars, 10 µm.

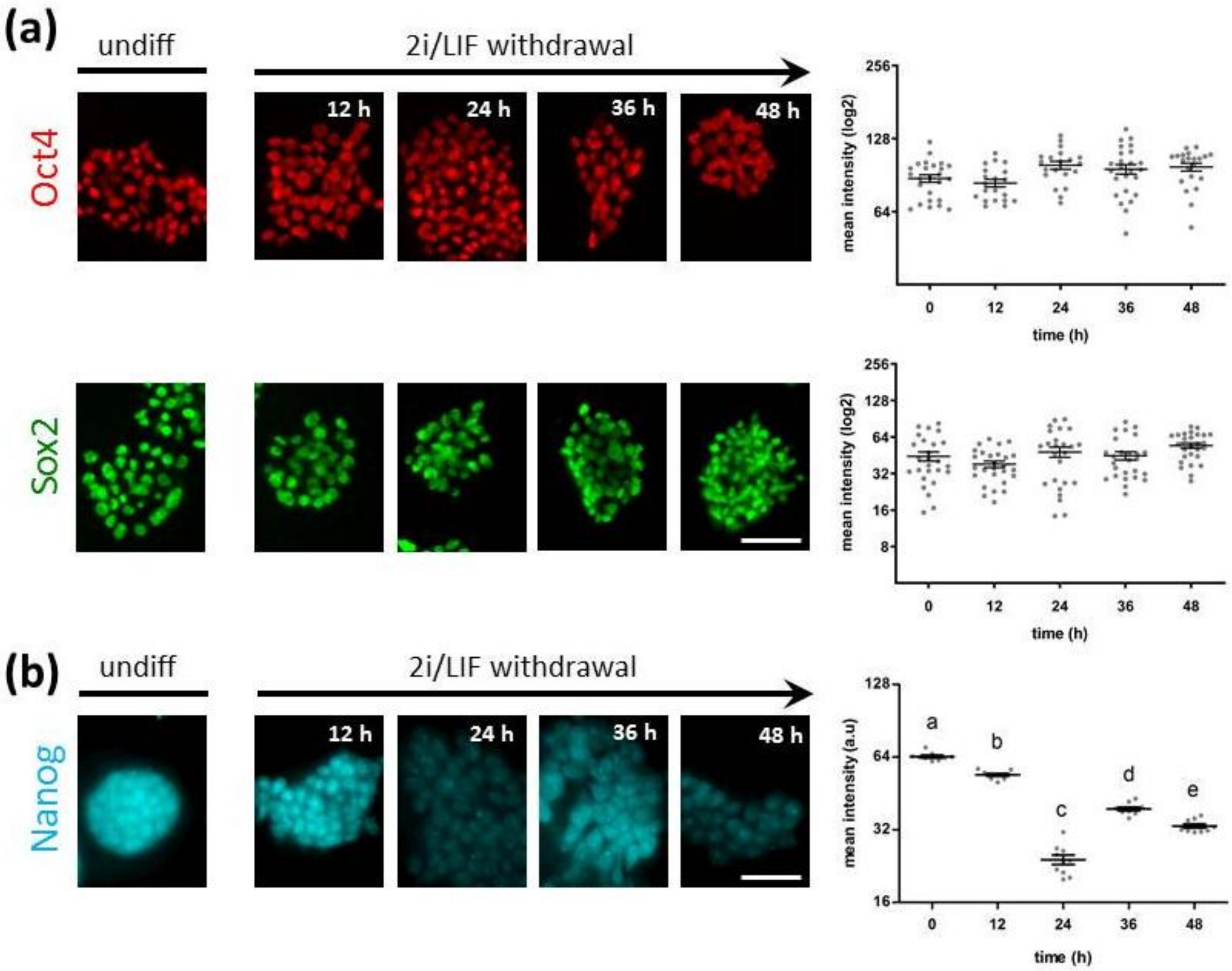

Figure S5. Oct4, Sox2 and Nanog levels in pluripotent ES cells and during early differentiation. Representative images of (a) Oct4, Sox2 and (b) Nanog immunofluorescence in undifferentiated (undiff) ES cells and at different times after 2i/LIF withdrawal. Scale bars, 60 µm. Dot plots represent the mean fluorescence intensity ± SEM. Each dot corresponds to a different image field. p-values were determined by ANOVA test and statistical comparisons among data were performed using a Tukey test. Different letters indicate significant differences ($p<0.01$).

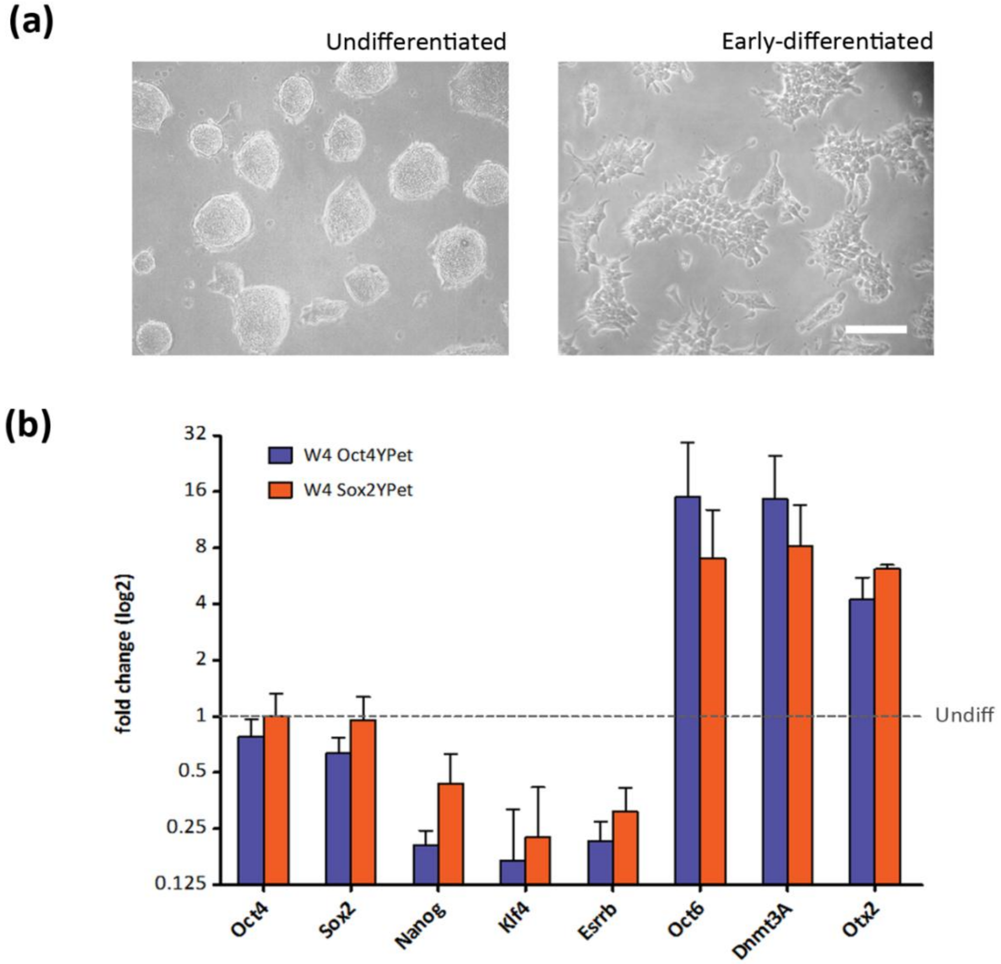

Figure S6. ES cells leave behind the ground state of pluripotency after 48 h of 2i/LIF withdrawal. (a) Representative images of Oct-YPet ES cells cultured with LIF +2i (undifferentiated) or after 48 h of LIF/2i withdrawal (early-differentiated). Scale bars: 100 µm. (b) mRNA levels of the indicated genes measured by RT-qPCR in Oct4-YPet and Sox2-YPet ES cells and relativized to those values obtained for the corresponding undifferentiated cells. Results are presented as mean ± SEM (n=2) and plotted in log2 scale.

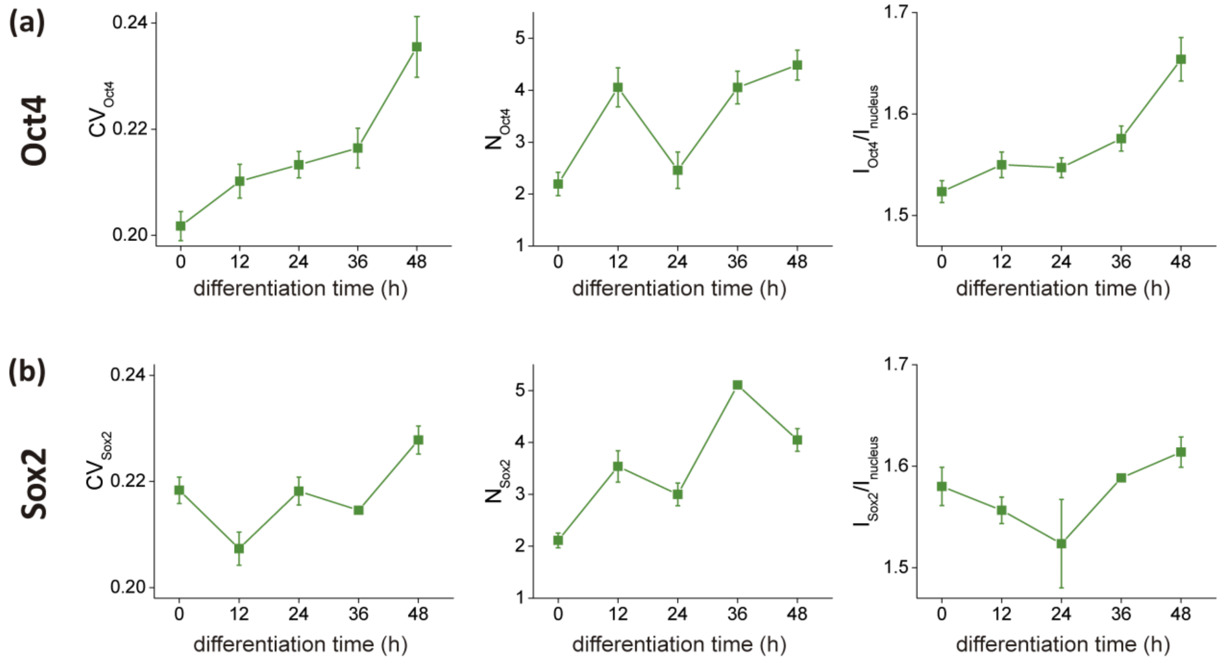

Figure S7. Reorganization of Oct4 and Sox2 during early differentiation assayed in independently generated clones. Coefficient of variation (CV), number of dense domains (N) and their intensity relative to the mean nuclear intensity ($I/I_{nucleus}$) for independently generated clones of Oct4-YPet **(a)** and Sox2-YPet **(b)** during the initial 48 h of differentiation induced by 2i/LIF withdrawal. The differences in the absolute parameter values with respect to those observed in Fig. 2 could be due to subtle variations within clones or, most probably, to slightly different conditions of the measurements. Results are presented as means ± SEM, N_{cells} was 40 for every time point determined for Oct4-YPet and 57, 40, 40, 40 and 61 for 0, 12, 24, 36 and 48 h, respectively for Sox2-YPet measurements.

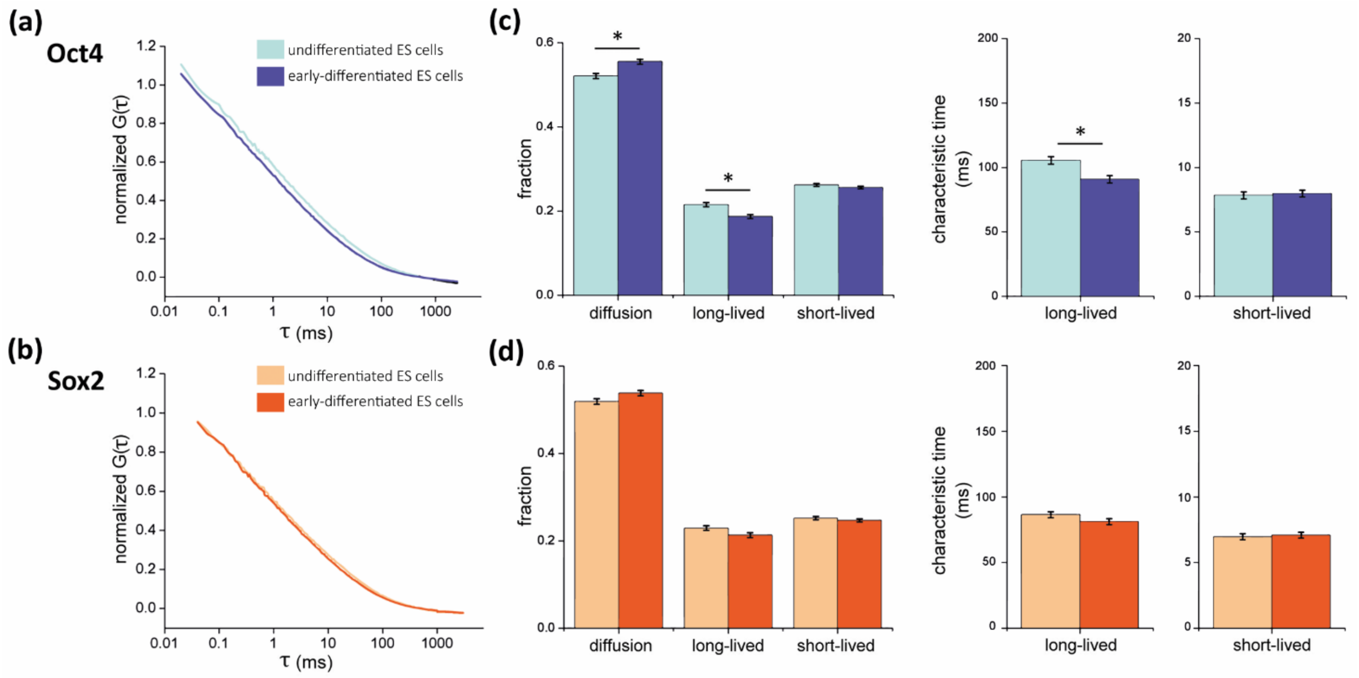

Figure S8. Dynamics of Oct4-YPet (a) and Sox2-YPet (b) in the nucleoplasm of cells corresponding to independently generated Oct4-YPet and Sox2-YPet clones. FCS measurements were run in undifferentiated independently generated clones of Oct4-YPet and Sox2-YPet ES cells (light colors) or 48 h after LIF/2i withdrawal (early-differentiated, dark colors). **(a, b)** Mean, normalized ACF obtained at the nucleoplasm. **(c, d)** The ACF data were fitted with Eq. 1 to obtain the fractions of free, long-lived bound and short-lived bound TFs and the characteristic times of long-lived and short-lived interactions of the TFs with chromatin. Experimental results were expressed as mean ± SEM ($n_{data\ Oct4}$ = 78 and 85 and $n_{data\ Sox2}$ = 65 and 64 for the undifferentiated and early-differentiated conditions, respectively). We only run a single experiment in each cell to minimize its photodamage. The differences in the characteristic time values between clones could be due to subtle variations within clones or, most probably, to slightly different conditions of the measurements. We only run a single experiment in each cell to minimize its photodamage. Asterisks (*) indicate significant differences ($p<0.01$) between undifferentiated and early-differentiated ES cells.

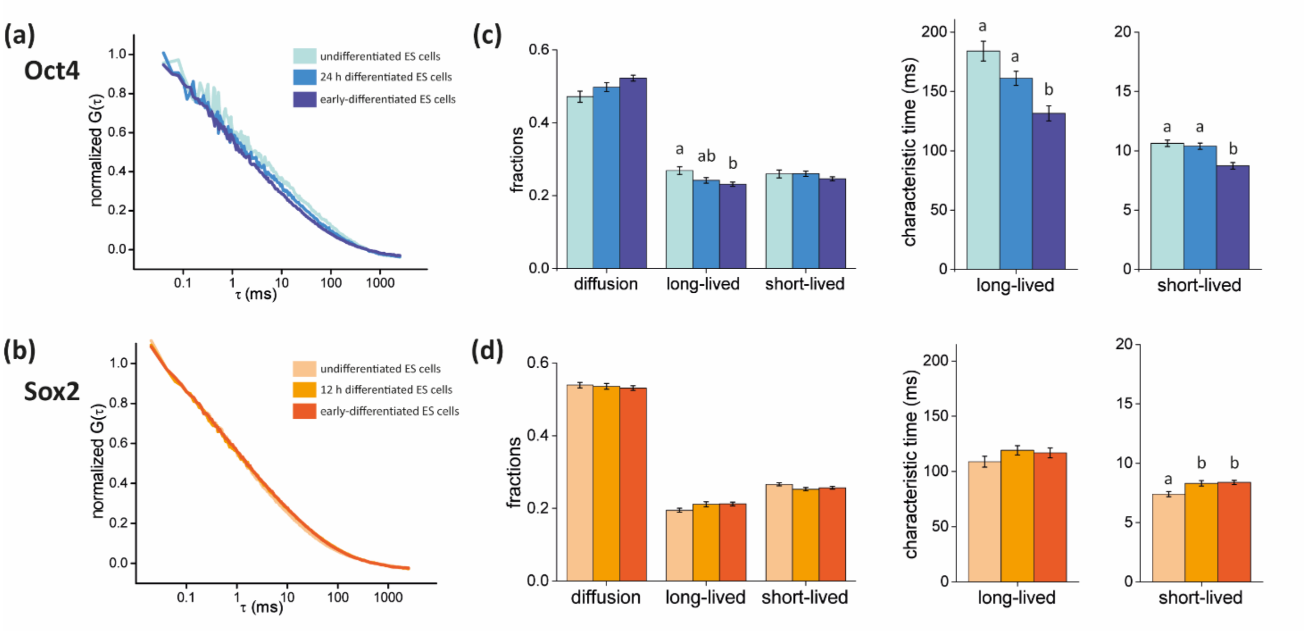

Figure S9. Dynamics of Oct4-YPet and Sox2-YPet at 24 and 12 h of differentiation. Single-point FCS measurements were run in Oct4-YPet **(a)** and Sox2-YPet **(b)** ES cells after 24 and 12 h of LIF/2i withdrawal, respectively. **(a, b)** Comparison of the mean, normalized ACF obtained after these differentiation periods with those obtained for undifferentiated and early-differentiated cells. **(c, d)** The ACF data were fitted with Eq. 1 to obtain the fractions of free, long-lived bound and short-lived bound TFs and the characteristic times of long-lived and short-lived interactions of the TFs with chromatin. The parameter values obtained after 24 or 12 h of differentiation (intermediate colors in each color-scale) are compared with those showed in Figure 3 for undifferentiated and early-differentiated cells. Experimental results were expressed as mean ± SEM (n_{data} = 61 and 81 for the intermediate time points studied for Oct4 and Sox2, respectively). We only run a single experiment in each cell to minimize its photodamage. Undifferentiated and early-differentiated data showed in Figure 3 are also included for a better comprehension. Different letters indicate significant differences ($p<0.01$).

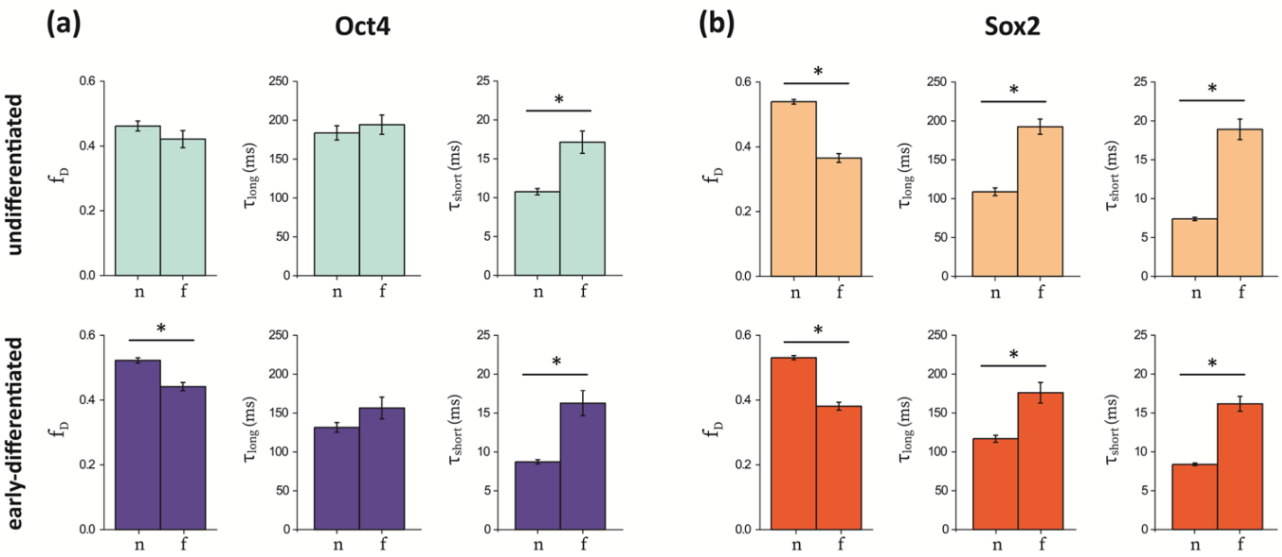

Figure S10. FCS reveals differences on Oct4 and Sox2 interactions with chromatin between nucleoplasm and foci. Single-point FCS measurements were run in undifferentiated Oct4-YPet **(a)** and Sox2-YPet **(b)** ES cells (light colors, top panels) or 48 h after LIF/2i withdrawal (dark colors, bottom panels). The ACF data were fitted with Eq. 1 to obtain the freely diffusing population fraction f_D and the residence times τ_{long} and τ_{short}. Experimental results were expressed as mean ± SEM. Asterisks (*) indicate significant differences ($p<0.01$) between nucleoplasm (n) and *foci* (f).

Supplementary Methods

Western blot analysis

Cells were trypsinized, collected by centrifugation and washed in ice-cold PBS. Then, the collected cells were lysed with RIPA buffer (50 mM Tris–HCl pH 8.0, 1% NP-40, 0.5% Sodium Deoxycholate, 0.1% SDS, 150 mM NaCl) containing protease inhibitors cocktail (Roche, Cat #11836170001). Lysis was allowed to proceed for 30 min on ice and further centrifuged for 20 min (4°C, 14,000 rpm) to separate soluble nuclear proteins from debris.

Protein samples were resolved on 10% SDS-polyacrylamide gels and transferred to PVDF membranes (Amersham). Membranes were blocked for 1 h at room temperature with 1% milk in PBS-T (blocking solution) and primary antibodies were incubated overnight at 4°C in blocking solution. Secondary antibodies were incubated for 1 h at room temperature. Membranes were revealed with ECL Prime Western Blotting Detection (GE Healthcare) in an Amersham Imager 600 System (Amersham). Antibodies and dilutions used are listed in the Supplementary Table S1.

Supplementary Tables

Table S1: Antibodies used for immunofluorescence (IF) and Western blot (WB)

Name	Company	Catalog #	Dilution
Nanog	Peprotech	500-P236	1:250 (IF)
Oct4	Santa Cruz	SC-5279	1:100 (IF), 1:400 (WB)
Sox2	Santa Cruz	SC-17320	1:500 (IF), 1:400 (WB)
SSEA-1	Santa Cruz	SC- 21702	1:250 (IF)
GFP	Affinity-purified	[1]	1:1000 (WB)
Anti-mouse AlexaFluor 488	Invitrogen	A21202	1:2000 (IF)
Anti-goat AlexaFluor 555	Invitrogen	A21432	1:2000 (IF)
Anti-rabbit AlexaFluor 488	Invitrogen	A21206	1:2000 (IF)
Anti-mouse IgG-HRP	Invitrogen	G-21040	1:1500 (WB)
Anti-goat IgG-HRP	Santa Cruz	SC-2020	1:1500 (WB)
Anti-rabbit IgG-HRP	Santa Cruz	SC-2357	1:1500 (WB)

Table S2: RT-qPCR primer sequences

Name	Note	Sequence (5'-3')
GAPDH	Forward	TGCCAAGGCTGTGGGCAAGG
	Reverse	CGAAGGTGGAAGAGTGGG
PGK1	Forward	TGGGCAAGGATGTTCTGTTC
	Reverse	TGCAGTCCCAAAAGCATCAT
Sox2	Forward	CACAACTCGGAGATCAGCAA
	Reverse	CTCCGGGAAGCGTGTACTTA
Nanog	Forward	AGGGTCTGCTACTGAGATGCTCTG
	Reverse	CAACCACTGGTTTTTCTGCCACCG
Oct4	Forward	TGACGGGAACAGAGGGAAAG
	Reverse	TCAGCTTGGGCTAGAGAAGG
YPet	Forward	ACCTACGGCGTGCAGTGCTT
	Reverse	TGCCGTCCTCCTTGAAGTCG

Supplementary References

1 Peche, L. Y. *et al.* Human MageB2 Protein Expression Enhances E2F Transcriptional Activity, Cell Proliferation, and Resistance to Ribotoxic Stress. *J Biol Chem* **290**, 29652-29662 (2015).

2 Buntru, A., Zimmermann, T. & Hauck, C. R. Fluorescence resonance energy transfer (FRET)-based subcellular visualization of pathogen-induced host receptor signaling. *BMC Biol* **7**, 81 (2009).

3 Pedersen, S. M., Chan, W., Jattani, R. P., Mackie d, S. & Pomerantz, J. L. Negative Regulation of CARD11 Signaling and Lymphoma Cell Survival by the E3 Ubiquitin Ligase RNF181. *Molecular and cellular biology* **36**, 794-808 (2015).

4 Chakraborty, A., Boel, N. M. & Edkins, A. L. HSP90 Interacts with the Fibronectin N-terminal Domains and Increases Matrix Formation. *Cells* **9** (2020).

5 Nguyen, A. W. & Daugherty, P. S. Evolutionary optimization of fluorescent proteins for intracellular FRET. *Nat Biotechnol* **23**, 355-360 (2005).

6 Shaner, N. C., Patterson, G. H. & Davidson, M. W. Advances in fluorescent protein technology. *J Cell Sci* **120**, 4247-4260 (2007).
